# In Vitro Propagation of *Gastrochilus matsuran* (Makino) Schltr., an Endangered Epiphytic Orchid

**DOI:** 10.3390/plants9040524

**Published:** 2020-04-18

**Authors:** Hyeonjeong Kang, Kyung Won Kang, Doo Hwan Kim, Iyyakkannu Sivanesan

**Affiliations:** 1 Babo Orchid Farm, Namyangju-si, Gyeonggi-do 472-831, Korea; sweetepik@naver.com (H.K.); dawin7@hanmail.net (K.W.K.); 2 Department of Bioresources and Food Science, Institute of Natural Science and Agriculture, Konkuk University, 1 Hwayang-dong, Gwangjin-gu, Seoul 05029, Korea; kimdh@konkuk.ac.kr

**Keywords:** asymbiotic seed germination, protocorm, auxin, gibberellic acid, thidiazuron, coconut water, Orchidaceae

## Abstract

*Gastrochilus matsuran* (Makino) Schltr. (Orchidaceae) populations are declining quickly because of overexploitation, climatic changes, and deforestation; therefore, mass-production protocols are required for this orchid. Natural propagation of this species is often hampered by meager seed germination and slow growth. Thus, our aim was to establish an effective protocol for the in vitro propagation of *G. matsuran* and reduce the risk of its extinction. We investigated the impacts of culture media, coconut water (CW), and plant hormones (gibberellic acid (GA_3_), indole-3-acetic acid (IAA), indole-3-butyric acid (IBA), α-naphthaleneacetic acid (NAA), and thidiazuron (TDZ)) on asymbiotic germination, multiplication and conversion of protocorms, and plantlet development. Maximal seed germination (93.3%) was achieved on ½ MS medium without vitamins plus 5% CW, 1 µM NAA, and 1.5 µM GA_3_. Secondary protocorm formation was best achieved on ½ MS medium without vitamins plus 2 µM TDZ. The conversion of protocorms into seedlings was maximized by supplementation with 2 µM IBA or 1 µM NAA. Acclimatized plantlets that exhibited exuberant growth on sphagnum moss were reintroduced to tree trunks in a natural habitat, with a 67% survival rate. This in vitro propagation procedure would be helpful for the mass production and conservation of this rare epiphytic orchid.

## 1. Introduction

*Gastrochilus matsuran* (Makino) Schltr. (Orchidaceae), known as the purple-spotted gastrochilus, is a miniature epiphytic orchid native to Japan, Korea, and Taiwan. In Korea, *G. matsuran* is found on rocks and tree trunks in the low mountains of Gyeongsangnam-do and Jeju-do. It has a high economic value in the ornamental industry. The natural populations of *G. matsuran* are declining quickly because of overexploitation, climatic changes, and deforestation. Therefore, the epiphytic orchid has been designated as a rare and endangered species, and it is regionally protected by the law [1]. Natural propagation of orchids is frequently hindered by meager seed germination and slow growth [2]. Therefore, tissue culture is a viable alternative technique for large-scale multiplication and conservation of this endangered orchid. Although mass propagation of orchids has been achieved through adventitious shoot regeneration [3], multiple shoot induction [4], and somatic embryogenesis [5], extensive large-scale propagation through protocorm-like bodies, induced from various explants (including seeds), is often preferred by orchid researchers [6,7,8,9]. The in vitro asymbiotic seed germination technique has been effectively used for the conservation and feasible production of endangered orchids such as *Cypripedium lentiginosum* [10], *Gastrochilus calceolaris* [11], *Gastrochilus japonicus* [12], *Pecteilis radiata* [13], and *Thrixspermum japonicum* [14].

**Figure 1:**
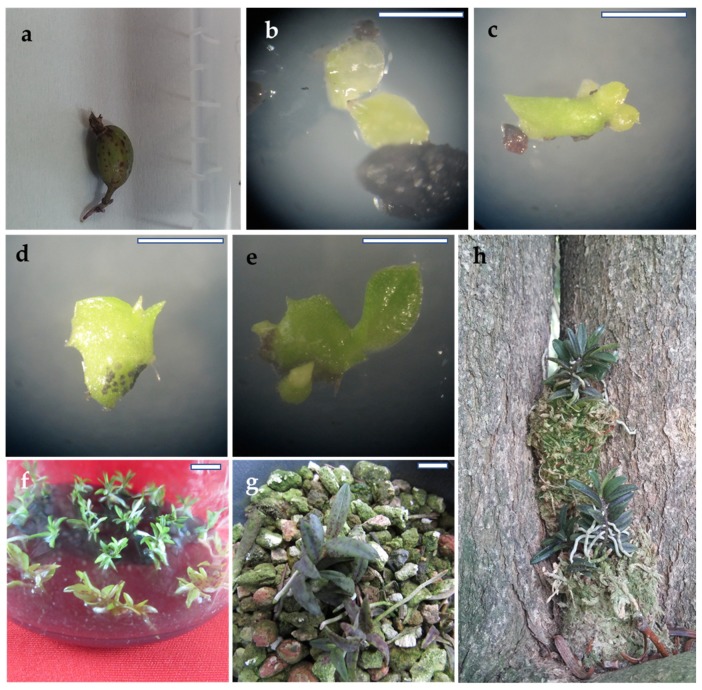
In vitro propagation of *G. matsuran*: (**a**) Seed capsule; (**b**) Induction of protocorm; (**c**) Induction of secondary protocorms; (**d**) Emergence of leaf primordia; (**e**) Elongation of leaf; (**f**) Seedlings development; (**g**) Plantlets acclimatized in the greenhouse; (**h**) Reintroduced acclimatized plantlets on the tree trunk. Scale bar: (a-e) 0.1 mm; (f and g) 1 cm.

Several group of factors, such as seed age, culture medium composition, environmental conditions, and genotype, influence the rate of asymbiotic in vitro seed germination [10]. The composition of the culture media is a significant factor that affects asymbiotic seed germination. Mineral nutrients, carbohydrates, vitamins, amino acids, growth hormones and organic acids are necessary for in vitro asymbiotic embryo development and protocorm formation [2]. Activated charcoal (AC), natural additives, and plant growth regulators (PGRs) are included in the culture media to enhance orchid seed germination and conversion of seedlings [2,11,12,13,14]. Successful procedures for in vitro regeneration of taxonomically related species such as *Gastrochilus calceolaris* [11] and *Gastrochilus japonicus* [12] have been reported. However, to the best of our knowledge, no information is available on the in vitro propagation of *G. matsuran*. In this study, we aimed to develop a procedure for the micropropagation of *G. matsuran* and reintroduction to its natural habitat. To establish a reliable and efficient procedure for mass propagation of *G. matsuran* and reduce the risk of its extinction, seeds from mature capsules (Figure 1a) were used to study the effects of growth media, coconut water (CW), and PGRs on the asymbiotic germination, multiplication and conversion of protocorms, and plantlet development of *G. matsuran* in vitro. Here, we have described an efficient technique for the rapid propagation of *G. matsuran*. This in vitro propagation procedure would be helpful for mass production as well as conservation of this rare epiphytic orchid.

## 2. Results

### 2.1. Impact of Culture Media on Seed Germination

**Figure 2:**
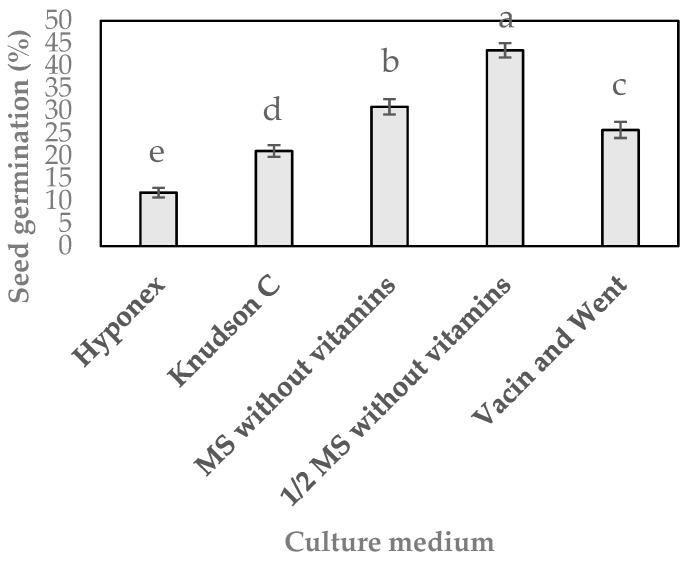
Effect of the culture medium on seed germination of *G. matsuran*. The culture media containing 0.05% activated charcoal, 1% banana pulp, 0.2% peptone, 3% sucrose, and 0.8% plant agar. Bars: ± standard error (SE). Letters (a-e) indicate differences among the growth media according to Duncan’s multiple range test (DMRT) at *p* ≤ 0.05.

Microbial contamination is one of the serious problems limiting the successful extrapolation of plant tissue culture practices. The successful initiation of in vitro culture mostly depends on surface sterilization of explants because this is the primary source. Numerous surface microflora atttached to plant surfaces, grow faster than the cultured explants and release phytotoxic substances into the culture media, hindering positive outcomes. The surface sterilization procedure produced 98% sterile *G. matsuran* seeds. Seeds isolated from the mature capsules of *G. matsuran* were inoculated on various media containing 0.05% AC, 1% banana pulp, 0.2% peptone, 3% sucrose, and 0.8% plant agar for germination. Asymbiotic in vitro seed germination of *G. matsuran* was affected significantly (*p* ≤ 0.05) by the culture medium (Figure 2). Seed germination was observed within seven weeks of incubation. After 10 weeks of culture, pale green protocorms were produced (Figure 1b). Germination percentages of 11.9%, 21.1%, 30.9%, 43.4%, and 25.8% were observed after 12 weeks of incubation on Hyponex, Knudson C, Murashige and Skoog (MS) without vitamins, ½ MS without vitamins, and Vacin and Went media, respectively (Figure 2). Among the five nutrient media, best seed germination rate was achieved on ½ MS (without vitamins) medium. Therefore, ½ MS (without vitamins) medium was used for the subsequent seed germination experiments.

### 2.2. Impact of CW and PGRs on Seed Germination

The asymbiotic seed germination rate of *G. matsuran* was affected significantly (*p* ≤ 0.05) by CW and PGRs. Supplementation of CW and PGRs (indole-3-acetic acid (IAA), α-naphthaleneacetic acid (NAA), and gibberellic acid (GA_3_) in ½ MS (without vitamins) medium containing 0.05% AC, 1% banana pulp, 0.2% peptone, 3% sucrose, and 0.8% plant agar increased the percentage of seed germination (Table 1). Among the four concentrations of CW tested, 5% induced the best (62%) seed germination. Therefore, this optimal CW level (5%) was used for the subsequent experiments. Of the four levels of IAA tested, 2 µM induced the best (72.8%) seed germination. Maximal seed germination (80.1%) was achieved when ½ MS (without vitamins) medium was amended with 1 µM NAA (Table 1). High levels of auxins, IAA and NAA, inhibited protocorm formation. Seed germination was affected significantly (*p* ≤ 0.05) by the GA_3_ level. The highest germination rate (93.3%) was achieved when ½ MS (without vitamins) medium plus 0.05% AC, 1% banana pulp, 0.2% peptone, 3% sucrose, 0.8% plant agar, 1 µM NAA was augmented with 1.5 µM GA_3_. However, the seed germination percentage significantly (*p* ≤ 0.05) decreased on media containing 2 and 3 µM GA_3_ (Table 1).

### 2.3. Impact of Thidiazuron (TDZ) on Secondary Protocorm Induction

The protocorm developed on 0.05% AC, 1% banana pulp, 0.2% peptone, 3% sucrose, 0.8% plant agar, 1 µM NAA and 1.5 µM GA_3_ was used for further studies. The optimal levels of banana pulp, and peptone for secondary protocorm formation were determined on the basis of the preliminary experiment. Secondary protocorms were observed on ½ MS (without vitamins) medium augmented with 0.05% AC, 1% banana pulp, 0.2% peptone, 3% sucrose, 0.8% plant agar and 1–8 µM TDZ (Figure 1c). The induction of secondary protocorms was affected significantly (*p* ≤ 0.05) by the TDZ level. Of the primary protocorms cultured on TDZ-free medium, 35.3% formed secondary protocorms, with a mean of 2.8 protocorms, after eight weeks of culture. After supplementation of TDZ at 1–8 µM, secondary protocorms were obtained in 43.3%–86.7% of the cultured primary protocorms (Table 2). The number of secondary protocorms varied from 3.7 to 8.3 per protocorm. TDZ at 2 µM yielded the best secondary protocorm induction (86.7%) and maximum (8.3) mean number of protocorms. High levels of TDZ (4 and 8 µM) resulted in a lower rate of protocorm formation (Table 2). On these media, protocorm development was inhibited, and browning of the protocorms was also observed.

### 2.4. Impact of Auxins on Protocorm Conversion and Seedling Development

**Figure 3:**
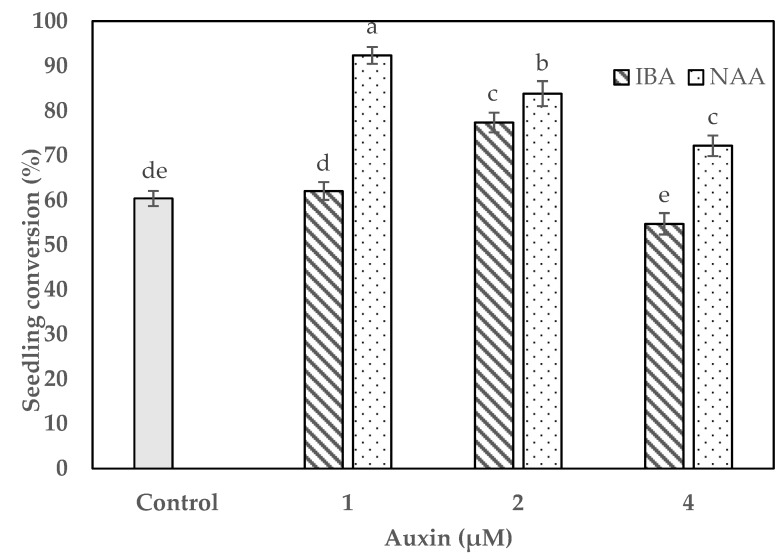
Effect of auxin on the conversion frequency of *G. matsuran* protocorms. Bars: ± SE. Letters (a-e) indicate differences among the treatments according to DMRT at *p* ≤ 0.05.

The conversion of secondary protocorms into seedlings and their development were investigated using various levels of natural additives. The optimal levels of banana pulp, peptone, and potato homogenate for protocorm conversion were determined on the basis of the preliminary experiment. Significant differences were detected in seedling conversion on ½ MS (with vitamins) medium with different concentrations of auxins (Figure 3). The protocorms differentiated on the conversion medium, developing shoot primordia, rhizoids, and new protocorm near the base within four weeks of culture (Figure 1d,e). After eight weeks of culture, the protocorms were converted into seedlings (Figure 1f). The conversion of protocorms into seedlings was maximized by the supplementation of optimal levels of indole-3-butyric acid (IBA; 2 µM) or NAA (1 µM). Nevertheless, increasing the optimal level of IBA or NAA could decrease the frequency of seedling conversion. The maximum seedling conversion rate was 92.3% for protocorms grown on ½ MS (with vitamins) medium containing 1 µM NAA (Figure 3).

### 2.5. Acclimatization and Reintroduction

**Figure 4:**
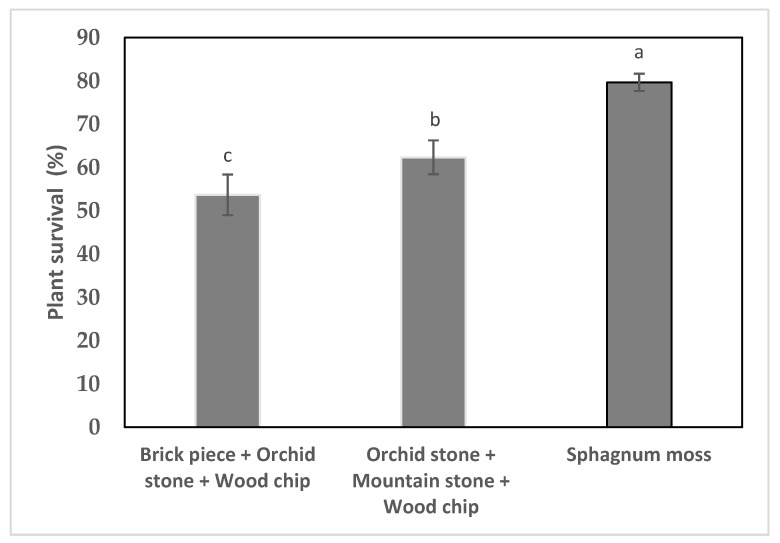
Survival of *G. matsuran* seedlings obtained in vitro six weeks after transfer to different substrates. Bars: ± SE. Letters (a-c) indicate differences among the substrates according to DMRT at *p* ≤ 0.05.

The seedlings grew well for four weeks after transplanting. The survival rate of the in vitro-raised *G. matsuran* was influenced by the substrates (Figure 4). About 53.7%–79.7% survival was achieved on brick pieces, orchid stone and wood chips, orchid stone, wood chips, and mountain stone or sphagnum moss after six weeks of transplanting. The highest rate of plant survival (79.7%) was observed on sphagnum moss (Figure 4). The lowest survival rate (53.7%) was observed on brick pieces + orchid stone + wood chips. However, the plantlets grew well on the potting mix (Figure 1g). Finally, plantlets that exhibited the best growth on sphagnum moss were reintroduced in tree trunks, and their growth and survival were monitored. The plantlets attached to the tree trunks grew well, with a survival rate of 67% (Figure 1h). The reintroduced plantlets were continuously monitored by our team to record seed capsule production.

## 3. Discussion

In vitro propagation of endangered plants is a potent technique for mass production and conservation, particularly for species with a diminished population. In general, symbiotic fungal infection is necessary for the germination of orchid seeds. The possibility of circumventing the fungal infection of orchid seeds during in vitro germination has added new aspects to orchid propagation [15]. Successful procedures for in vitro asymbiotic seed germination have been reported for numerous orchids [10,11,12,13,14]. However, the seed germination percentage depends primarily on the medium composition. In this study, seeds of *G. matsuran* were germinated on Hyponex, Knudson C, MS (without of vitamins), ½ MS (without of vitamins), and Vacin and Went media. However, the seeds germinated best on ½ MS (without of vitamins) medium (Figure 2). Similarly, Kim et al. [12] reported that seeds of *G. japonicus* germinated best in ½ MS (without of vitamins) medium. In contrast, Pathak et al. [11] reported that a higher percentage of *G. calceolaris* seeds germinated in vitro when placed on MS medium [11]. This indicates that the nutritional requirements for seed germination may vary among species. Several orchids prefer a low level of macro- and micro-elements for seed germination [2]. The basal medium is augmented by various natural additives and PGRs to boost orchid seed germination. In *G. japonicus* the maximal seed germination (95.4%) was observed in culture medium containing 50 mL/L CW. Pathak et al. [11] reported that seeds of *G. calceolaris* germinated best (99.5%) in MS medium containing either 1.0 g/L yeast extract or 10% CW. In this study, addition of CW to ½ MS (without of vitamins) medium increased the percentage of seed germination (72.8%) in *G. matsuran*. CW has also been reported to stimulate seed germination in *Coelogyne nervosa* A. Rich. [16], *Cymbidium* hybrid [17], *Cypripedium macranthos* Sw. [18], *Papilionanthe teres* (Roxb.) Schltr. [19], and *Vanda stangeana* Rchb. F. [20]. It contains organic acids, minerals, plant hormones, sugars, and vitamins, which appear to influence seed germination in orchids [18,21]. The addition of IAA or NAA to the basal medium either increased or decreased the rate of seed germination depending on their concentrations (Table 1). NAA had a better effect than IAA on the seed germination of *G. matsuran*. Similar results have been observed for *P. radiata* [13] and *T. japonicum* [14]. GA_3_ is an important PGR added to the basal medium to improve the rate of seed germination. It has been used to enhance seed germination in different orchids such as *Calanthe discolor* Lindl. [22], *Comparettia falcata* Poepp. and Endl. [23], and *Phalaenopsis* [24]. In this study, the highest seed germination (93.3%) was obtained on the medium augmented with 5% CW, 1 µM NAA, and 1.5 µM GA_3_.

TDZ is an important PGR that plays a vital role in in vitro micropropagation of orchids. It has been used to induce protocorms or protocorm-like bodies in different orchids such as *Ansellia africana* Lindl. [25], *C. lentiginosum* [10], *Cyrtopodium glutiniferum* Raddi. [26], *Dendrobium aqueum* Lindl. [27], *G. japonicus* [12], *P. radiata* [13], and *Vanda* hybrid ‛Robert’s Delight’ [28]. The addition of 1–8 µM TDZ to the culture medium significantly increased both the rate of protocorm induction and number of secondary protocorms when compared with the control medium (Table 2). Maximum secondary protocorms were formed on ½ MS (without of vitamins) medium augmented with 5% CW, 0.5 g L^-l^, and 2.0 µM TDZ. Increasing the TDZ level (4 and 8 µM) reduced the frequency of secondary protocorm induction and number of secondary protocorms in *G. matsuran*. Adverse effects of high TDZ concentrations on protocorm formation have also been reported in *A. africana* [25], *C. glutiniferum* [10], *G. japonicus* [12], and *P. radiata* [13].

Seedling conversion was achieved on ½ MS (with vitamins) medium augmented with various amounts of IBA or NAA. Of the two auxins used, NAA showed the best results for seedling conversion (Figure 3). Kim et al. [12] also reported that the supplementation of Hyponex medium with NAA improved the seedling conversion rate of *G. japonicus*. Auxins often promote orchid seed germination, protocorm conversion, and seedling development [13,14,29]. Acclimatization of in vitro-developed seedlings plays a crucial role in the large-scale propagation of various orchids. Although *G. matsuran*, a rare and endangered orchid of East Asia, usually grows on tree trunks in a natural habitat, many epiphytic orchids such as *G. calceolaris* [11], *G. japonicus* [12], *Nothodoritis zhejiangensis* Z.H.Tsi [30], *T. japonicum* [14], and *V. stangeana* [16] have been successfully cultivated in the greenhouse by using a mixture of different substrates such as brick pieces, fir bark blocks, pine bark, orchid stone, vermiculite, sphagnum moss, charcoal pieces, and coconut husk chips. Potting mix is plays an important role in plantlet survival during acclimatization. The substrate mixtures should provide the best conditions such as moisture retention, aeration and drainage for enhancing the survival rate of plantlets [12,13,14]. *Gastrochilus calceolaris* exhibited 70%–80% survival on a mixture of brick pieces, pine bark, sphagnum moss, and charcoal pieces (1:1:1:1) [11]. Kim et al. [12] achieved 86% survival of *G. japonicus* plantlets when they were transplanted into a pot filled with a layer of orchid stone (root zone), wood chips (middle), and mountain stone (top). In this study, the in vitro-developed *G. matsuran* seedlings were acclimatized in a greenhouse at a 53.7%–79.7% survival rate. The highest rate of plant survival was achieved on sphagnum moss. This effect might be due to adequate water and nutrients and availability to plants by sphagnum moss. The acclimatized plants adapted to the natural conditions and grew well.

## 4. Materials and Methods

### 4.1. Plant Materials and Surface Disinfection

Mature seed capsules of *G. matsuran* were collected from Jeju Island, Korea. The seed capsules were washed under tap water for 5–7 min. The seed capsules were surface-disinfected using ethanol (70%) for five minutes, maintained in sterilized double-distilled water for 60 s, followed by sodium hypochlorite solution (2%) for 15 min. The disinfected capsules were rinsed five times with sterilized double-distilled water. Finally, the seeds were excised from the disinfected capsules.

### 4.2. Impact of Culture Media on Seed Germination

The seeds were transferred to 500 mL culture bottles with 120 mL of Hyponex (N:P:K; 6.5:6:19) medium (3 g L^−l^), Knudson C medium [11], MS [31] (without vitamins), ½ MS (without vitamins) and Vacin and Went [32] media. All seed germination media were augmented with 0.05% AC, 1% banana pulp (obtained from banana cultivar Cavendish after homogenization in a blender), 0.2% peptone, 3% sucrose, and 0.8% plant agar (pH 5.7 ± 1). The media were autoclaved at 121 °C and 103.95 kPa for 20 min. The seed cultures were incubated at 22 ± 2 °C and 16 h light/8 h dark photoperiod (light provided by cool white fluorescent tubes at 10 µmol s^−1^ m^−2^). The number of spherules was counted at seven-day intervals by using a light microscope. The impact of culture media on the seed germination of *G. matsuran* was recorded after 12 weeks.

### 4.3. Impact of CW and PGRs on Seed Germination

To evaluate the effects of CW and PGRs on germination, the seeds of *G. matsuran* were placed on ½ MS (without vitamins) medium augmented with 0%, 2.5%, 5%, 7.5%, or 10% CW; 0, 0.5, 1, 2, or 4 µM IAA or NAA in combination with 5% CW; 0, 0.5, 1, 1.5, 2, or 3 µM GA_3_ in combination with 5% CW; and 1 µM NAA (pH 5.7 ± 1). The impact of CW and PGRs on the seed germination of *G. matsuran* was recorded after 12 weeks.

### 4.4. Impact of TDZ on Secondary Protocorm Induction

The protocorms developed from the germinated seeds of *G. matsuran* were transferred to ½ MS (without vitamins) medium augmented with 0, 1, 2, 4, or 8 µM TDZ for secondary protocorm formation. The protocorm induction medium was augmented with 0.05% AC, 1% banana pulp, 0.2% peptone, 3% sucrose, and 0.8% plant agar (pH 5.7 ± 1). The protocorm cultures were maintained at 22 ± 2 °C and 16 h light/8 h dark photoperiod (light provided by cool white fluorescent tubes at 45 µmol s^−1^ m^−2^). The secondary protocorm induction percentage and mean protocorm number were assessed after eight weeks.

### 4.5. Impact of IBA and NAA on Protocorm Conversion and Seedling Development

The secondary protocorms were subcultured on ½ MS (with vitamins) medium with 0, 1, 2, or 4 µM IBA or NAA for the conversion of seedlings. The seedling conversion medium was augmented with 0.05% AC, 1% banana pulp, 0.2% peptone, 2% potato homogenate, 3% sucrose, and 0.8% plant agar (pH 5.7 ± 1). The protocorm cultures were maintained at 22 ± 2 °C and 16 h light/8 h dark photoperiod (light provided by cool white fluorescent tubes at 45 µmol s^−1^ m^−2^). The seedling conversion percentage was assessed after 12 weeks of incubation.

### 4.6. Acclimatization and Reintroduction

Well-developed seedlings (16 weeks of age) isolated from the NAA treatments were washed under tap water for a few minutes to remove excess agar from the roots. The seedlings were transplanted into plastic pots containing a mixture of brick pieces, orchid stone, and wood chips (1:1:1), a layer of orchid stone (root zone), wood chips (middle), and mountain stone (top) or sphagnum moss inside a greenhouse. The pots were covered with transparent plastic covers with three holes to maintain relative humidity. After 10 days, the transparent plastic covers were removed, and the pots were incubated under 60% shade for four weeks in the greenhouse. The seedlings were watered every other day and fertilized with Hyponex (N:P:K; 20:20:20) solution (2 g L^−1^) at 14-day intervals. Survival was recorded after six weeks. The well-established plantlets cultivated in the greenhouse were reintroduced to the tree trunks of *Acer palmatum, Pinus densiflora* and *Torreya nucifera* at Jeju Island, Korea in spring. For each treatment, 25 plantlets were used. Survival was recorded after five weeks.

### 4.7. Statistical Analysis

All the experiments were performed twice. Each seed germination treatment consisted of 10 replications (bottles), with each bottle containing more than 200 seeds; each secondary protocorm initiation treatment consisted of 10 replications (bottles), with each bottle containing 25 protocorms; each seedling conversion treatment consisted of 15 replications (bottles), with each bottle containing 20 protocorms; and each acclimatization treatment consisted of 10 replications, and for each replicate, 25 seedlings were used. The obtained data were subjected to analysis of variance by using the SAS program (Release 9.4, SAS Institute, NC, USA). Mean comparison was achieved with DMRT (*p* ≤ 0.05).

## 5. Conclusions

To the best of our knowledge, this is the first report of an efficient mass production method for the micropropagation of *G. matsuran*. Overall, ½ MS (without vitamins) with 5% CW, 1 µM NAA, and 1.5 µM GA_3_; ½ MS (without vitamins) with 5% CW and 2 µM TDZ; and ½ MS (w vitamins) with 1 µM NAA were the best media for the seed germination, protocorm multiplication, and seedling conversion of *G. matsuran*, respectively. The seedlings acclimatized in the greenhouse were successfully reintroduced to tree trunks in a natural habitat.

## Figures and Tables

**Table 1:** Effect of coconut water and plant growth regulators on seed germination of *G. matsuran*.

CW (%)	IAA (µM)	NAA (µM)	GA_3_ (µM)	Seed Germination (%)
0	-	-	-	43.4 ± 1.6 i
2.5	-	-	-	53.9 ± 2.3 gh
5.0	-	-	-	62.0 ± 3.0 ef
7.5	-	-	-	57.2 ± 2.2 fg
10.0	-	-	-	47.8 ± 2.0 hi
5.0	0.5	-	-	58.3 ± 3.1 fg
5.0	1.0	-	-	66.7 ± 2.4 de
5.0	2.0	-	-	72.8 ± 2.9 cd
5.0	4.0	-	-	51.4 ± 2.4 gh
5.0	-	0.5	-	67.9 ± 1.6 cde
5.0	-	1.0	-	80.1 ± 2.3 b
5.0	-	2.0	-	73.1 ± 2.4 bcd
5.0	-	4.0	-	49.2 ± 2.5 hi
5.0	-	1.0	1.0	87.8 ± 2.6 a
5.0	-	1.0	1.5	93.3 ± 1.6 a
5.0	-	1.0	2.0	75.2 ± 2.5 bc
5.0	-	1.0	3.0	69.9 ± 2.5 cd

Means ± SE within a column followed by the same letter (a-i) are not significantly different according to DMRT at *p* ≤ 0.05.

**Table 2:** Effect of thidiazuron on secondary protocorm induction.

TDZ(µM)	Secondary Protocorm Induction (%)	Number of Secondary Protocorms
0	35.3 ± 1.2 d	2.8 ± 0.4 c
1.0	59.3 ± 2.1 b	4.6 ± 0.6 b
2.0	86.7 ± 2.4 a	8.3 ± 0.6 a
4.0	64.9 ± 1.7 b	5.1 ± 0.7 b
8.0	43.3 ± 2.2 c	3.7 ± 0.7 bc

Means ± SE within a column followed by the same letter (a-d) are not significantly different according to DMRT at *p* ≤ 0.05.

## References

[1] Kim J.Y., Do Y., Im R.-Y., Kim G.-Y., Joo G.-J. (2014). Use of large web-based data to identify public interest and trends related to endangered species. Biodivers. Conserv..

[2] Kauth P.J., Dutra D., Johnson T.R., Stewart S.L., Kane M.E., Vendrame V., da Silva J.A.T. (2008). Techniques and applications of in vitro orchid seed germination. Floriculture, Ornamental and Plant Biotechnology: Advances and Topics Issues.

[3] Park H.Y., Kang K.W., Kim D.H., Sivanesan I. (2018). In vitro propagation of *Cymbidium goeringii* Reichenbach fil. through direct adventitious shoot regeneration. Physiol. Mol. Biol. Plants.

[4] Kaur S. (2017). In vitro regeneration of shoots from nodal explants of *Dendrobium chrysotoxum* Lindl. J. Hortic. Res..

[5] Shen H.J., Chen J.T., Chung H.H., Chang W.C. (2018). Plant regeneration via direct somatic embryogenesis from leaf explants of *Tolumnia Louise* Elmore ‘Elsa’. Bot. Stud..

[6] Bustam S., Sinniah U.R., Swamy M.K. (2017). Simple and efficient in vitro method of storing *Dendrobium* sw Shavin White protocorm like bodies (PLBs). Bangladesh J. Bot..

[7] Chookoh N., Chiu Y., Chang C., Hu W., Dai T. (2019). Micropropagation of *Tolumnia* orchids through induction of protocorm-like bodies from leaf segments. Hortscience.

[8] Mehraj H., Alam M.M., Habiba S.U., Mehbub H. (2019). LEDs combined with CHO sources and CCC priming PLB regeneration of *Phalaenopsis*. Horticulture.

[9] Cardoso J.C., Zanello C.A., Chen J.-T. (2020). An overview of orchid protocorm-like bodies: Mass propagation, biotechnology, molecular aspects, and breeding. Int. J. Mol. Sci..

[10] Jiang H., Chen M.-C., Lee Y.-I. (2017). In vitro germination and low-temperature seed storage of *Cypripedium lentiginosum* P.J. Cribb & S.C. Chen, a rare and endangered lady’s slipper orchid. Sci. Hortic..

[11] Pathak P., Piri H., Vij S.P., Mahant K.C., Chauhan S. (2011). In vitro propagation and mass scale multiplication of critically endangered epiphytic orchid, *Gastrochilus calceolaris* (Buch.-ham ex. J.Sm.) D. Don using immature seeds. Indian J. Exp. Biol..

[12] Kim D.H., Kang K.W., Sivanesan I. (2019). In vitro germination and seedling development of *Gastrochilus japonicus* (Makino) Schltr. Propag. Ornam. Plants.

[13] Kim D.H., Kang K.W., Enkhtaivan G., Jan U., Sivanesan I. (2019). Impact of activated charcoal, culture medium strength and thidiazuron on non-symbiotic in vitro seed germination of *Pecteilis radiata* (Thunb.) Raf. S. Afr. J. Bot..

[14] Seon K.M., Kim D.H., Kang K.W., Sivanesan I. (2018). Highly competent in vitro propagation of *Thrixspermum japonicum* (Miq.) Rchb.f., a rare epiphytic orchid. In Vitro Cell. Dev. Biol. Plant.

[15] Knudson L. (1946). A new nutrient solution for germination of orchid seed. Am. Orchid. Soc. Bull..

[16] Abraham S., Augustine J., Thomas T.D. (2012). Asymbiotic seed germination and in vitro conservation of *Coelogyne nervosa* A. Rich. an endemic orchid to western Ghats. Physiol. Mol. Biol. Plants.

[17] Kim D.H., Kang K.W., Sivanesan I. (2017). In vitro propagation of *Cymbidium* hybrid. Propag. Ornam. Plants.

[18] Huh Y.S., Lee J.K., Nam S.Y., Paek K.Y., Suh G.U. (2016). Improvement of asymbiotic seed germination and seedling development of *Cypripedium macranthos* Sw. with organic additives. J. Plant Biotechnol..

[19] Zhou X., Gao J.Y. (2016). Highly compatible Epa-01 strain promotes seed germination and protocorm development of *Papilionanthe teres* (Orchidaceae). Plant Cell Tissue Organ Cult..

[20] Bembemcha P., Kishor R., Bai N. (2016). In vitro immature embryo germination and propagation of *Vanda stangeana* Rchb. f., an orchid endemic to India. Hortic. Environ. Biotechnol..

[21] Molnár Z., Virág E., Ördög V. (2011). Natural substances in tissue culture media of higher plants. Acta Biol. Szeged..

[22] Miyoshi K., Mii M. (1995). Phytohormone pretreatment for the enhancement of seed germination and protocorm formation by the terrestrial orchid, *Calanthe discolor* (Orchidaceae), in asymbiotic culture. Sci. Hortic..

[23] Manrique J.P., Fernandex-Lizarazo C., Suarez-Silva A. (2005). Evaluation of the effect of three growth regulators in the germination of *Comparetia falcate* seeds under in vitro conditions. In Vitro Cell. Dev. Biol. Plant.

[24] Heirati H.K., Onsinejad R., Yari F. (2015). The effect of pollination time and gibberellic acid (GA_3_) on the production and seed germination of *Phalaenopsis* orchids. J. Ornam. Plants.

[25] Bhattacharyya P., Kumar V., Grúz J., Doležal K., Van Staden J. (2019). Deciphering the phenolic acid reserves and antioxidant activity within the protocorm like bodies of *Ansellia africana*: A vulnerable medicinal orchid. Ind. Crops Prod..

[26] Vogel I.N., Macedo A.F. (2011). Influence of IAA, TDZ, and light quality on asymbiotic germination, protocorm formation, and plantlet development of *Cyrtopodium glutiniferum* Raddi, a medicinal orchid. Plant Cell Tissue Organ Cult..

[27] Parthibhan S., Rao M.V., Kumar T.S. (2015). In vitro regeneration from protocorms in *Dendrobium aqueum* Lindley—An imperiled orchid. J. Genet. Eng. Biotechnol..

[28] Korah P.L., Shylaraj K.S. (2018). In vitro proliferation competence of protocorm-like-bodies for direct induction of shoot buds for multiplication of *Vanda* ‘Robert’s Delight. Propag. Ornam. Plants.

[29] Novak S.D., Luna L.J., Gamage R.N. (2014). Role of auxin in orchid development. Plant Signal. Behav..

[30] Zeng S.J., Chen Z.L., Wu K.L., Zhang J.X., Bai C.K., Teixeira da Silva J.A., Duan J. (2011). Asymbiotic seed germination, induction of calli and protocorm-like bodies, and in vitro seedling development of the rare and endangered *Nothodoritis zhejiangensis* Chinese orchid. HortScience.

[31] Murashige T., Skoog F. (1962). A revised medium for rapid growth and bioassays with tobacco tissue cultures. Physiol. Plant..

[32] Vacin E., Went F. (1949). Some pH changes in nutrient solution. Bot. Gazette.

